# Serum 25–Hydroxyvitamin D_3_ and Mammography Density among Mexican Women

**DOI:** 10.1371/journal.pone.0161686

**Published:** 2016-08-26

**Authors:** Amina Amadou, Carine Biessy, Sabina Rinaldi, Veronika Fedirko, Nada Assi, Martin Lajous, Eduardo Ortiz-Panozo, Elsa Yunes, Ruy Lopez-Ridaura, Gabriela Torres-Mejia, Isabelle Romieu

**Affiliations:** 1 Nutrition and Metabolism Section, International Agency for Research on Cancer (IARC), Lyon, France; 2 Department of Epidemiology, Rollins School of Public Health, Winship Cancer Institute, Emory University, Atlanta, GA, United States of America; 3 Center for Research on Population Health, National Institute of Public Health, Cuernavaca, Mexico; 4 Department of Epidemiology, Harvard School of Public Health, Boston, MA, United States of America; University of North Dakota School of Medicine and Health Sciences, UNITED STATES

## Abstract

Low circulating levels of vitamin D and high mammographic density (MD) have been associated with higher risk of breast cancer. Although some evidence suggested an inverse association between circulating vitamin D and MD, no studies have investigated this association among Mexican women. We examined whether serum 25−hydroxyvitamin D3 [25(OH)D3] levels were associated with MD in a cross-sectional study nested within the large Mexican Teacher's Cohort. This study included 491 premenopausal women with a mean age of 42.9 years. Serum 25(OH)D3 levels were measured by liquid chromatography/tandem mass spectrometry. Linear regression and non-linear adjusted models were used to estimate the association of MD with serum 25(OH)D3. Median serum 25(OH)D3 level was 27.3 (23.3–32.8) (ng/ml). Forty one (8%) women had 25(OH)D3 levels in the deficient range (< 20 ng/ml). Body mass index (BMI) and total physical activity were significantly correlated with 25(OH)D3 (r = −0.109, P = 0.019 and r = 0.095, P = 0.003, respectively). In the multivariable linear regression, no significant association was observed between 25(OH)D3 levels and MD overall. However, in stratified analyses, higher serum 25(OH)D3 levels (≥27.3 ng/ml) were significantly inversely associated with percent MD among women with BMI below the median (β = −0.52, P = 0.047). Although no significant association was observed between serum 25(OH)D3 and percent MD in the overall population, specific subgroups of women may benefit from higher serum 25(OH)D3 levels.

## Introduction

Mammographic density (MD) has been identified among the strongest predictors of breast cancer (BC) risk. Women having more than 75% of dense tissue have 4 to 6 times greater risk of BC compared to women with little dense tissue [1–3]. MD represents the dense tissue of the breast, and is expressed as a percentage. Women with high MD have more proliferating epithelial tissue and connective tissue relative to women with low MD [4, 5]. Proliferating cells are more vulnerable to genetic damage, thereby increasing BC risk [6]. MD is correlated with several BC risk factors including age, anthropometry, reproductive, genetic, and hormonal factors, diet, and circulating micronutrients [5, 7], including low vitamin D levels [8–10].

Vitamin D is a fat-soluble vitamin that is naturally present in very few foods, but is available as dietary supplements, or produced in the skin in response to ultraviolet B (UVB) exposure [11, 12]. Vitamin D3 (cholecalciferol) is the precursor to the steroid hormone calcitriol, and it is activated to its active form by two cytochrome P450-mediated hydroxylation steps [13]. The first hydroxylation step mostly occurs in the liver to yield 25-hydroxvitamin D3 (25(OH)D3), which is catalyzed by the enzyme vitamin D-25-hydroxylase (predominantly CYP2R1). 25(OH)D3 is the circulating form of the hormone that is measured in the blood and clinically used to establish and monitor the vitamin D status of a patient. Circulating 25(OH)D3 is hydroxylated in the kidney by the cytochrome P450 enzyme CYP27B1 (1α-hydroxylase) to yield calcitriol [13, 14]. Vitamin D2 (ergocalciferol) is a form of vitamin D that is of plant origin, is derived from ergosterol and functions much like vitamin D3 but is less active [14]. Serum 25(OH)D (25(OH)D3 and 25(OH)D2) concentration is the major circulating form of vitamin D, and the best biomarker reflecting exposure to vitamin D from different sources, with a half-life of 2-3weeks [12, 15]. Studies have shown that vitamin D can promote apoptosis and cell differentiation, and inhibits breast cell proliferation that may have an effect on estrogen metabolism [14, 16].

There are a number of studies on the association between vitamin D (estimated from the diet, or measured as biomarker) and MD among both pre and postmenopausal women [8, 17–24]. Although some of them consistently reported significant inverse associations between vitamin D and MD [18–20, 24], the evidence of associations continue to be inconsistent [21–23, 25] and to this date, no study has investigated this association among Mexican women.

It is hypothesized that the actions of vitamin D, calcium, insulin-like growth factor (IGF)-I, and IGF-binding protein-3 (IGFBP-3) on BC are interconnected [26]. IGF-I levels have been positively related to MD among premenopausal Canadian women while IGFBP-3 levels have been inversely related [27]. Both Epidemiological and molecular studies have shown that vitamin D, directly or indirectly, may inhibit IGF-I and enhance IGFBP-3 effects in breast tissue, that may reduce breast density and breast cancer risk [16, 28]. IGF1-stimulated cell growth was inhibited by vitamin D analogs and this effect was associated with increased release of IGF binding protein 3 (IGFBP3) [29]. Deeb et al demonstrated that vitamin D treatment can result in the upregulation of IGFBP3 and transforming growth factor‑β (TGF β)–SMAD3 signaling cascades and by downregulating the epidermal growth factor receptor (EGFR) signaling pathway [16]. Further evidence reported that, in breast tumours, vitamin D modulates the IGF-I/IGFBP ratio to decrease proliferation and increase apoptosis [30]. However, there is no data on these associations in the Hispanic population that might help better understand mechanisms by which vitamin D may affect MD and BC.

To further clarify the role of vitamin D in MD, we examined whether serum 25(OH)D levels were associated with MD, and whether this association differed by obesity status in a cross-sectional study nested within the Mexican Teachers’ Cohort (MTC). A second objective was to explore the modifying effect and mediating role of IGF pathway (as IGF1 and IGFP3) in the association between 25(OH)D and MD.

## Materials and Methods

### Study population

The Mexican Teachers’ Cohort (MTC) is a prospective study of 115,315 female teachers from 12 Mexican states aged 35 years who were invited to participate in a cohort study to evaluate lifestyle and chronic diseases. Detailed methods have been described elsewhere [31]. Information were obtained on socio-demographics, socio-economic status (SES), reproductive history, hormone contraceptive and menopausal hormone replacement therapy use, physical activity, alcohol consumption, smoking history, family history of breast cancer (FHBC), clinical history, and lifestyle (including a food frequency questionnaire). In 2007, a subsample of 2,084 MTC participants from two Mexican regions (Jalisco and Veracruz) participated in a clinical evaluation that included an interview, anthropometric measurements, mammogram and the collection of biological specimens. Fasting blood samples (~25 ml) were obtained through venipuncture by trained nurses.

### Selection of subjects

Among the 2,084 participants who participated in the clinical sub-cohort, we excluded 230 women who had insufficient information on metabolic syndrome components (because of a parallel study on metabolic syndrome in the same population [32], 67 who had an unknown menopausal status and 624 who were postmenopausal at the time of their mammogram. Women were considered as pre-menopausal if they had menstruated at least once over the 12 months prior to the visit, and were considered as postmenopausal if they had no menstruation over the last 12 months prior to the visit, and those with surgical menopause who reported bilateral oophorectomy [33]. We then stratified women by 4 breast density categories: <10%, 10 to <25%, 25 to <50% and > = 50% [34]. Women were randomly selected from each group proportionally to its size. Thirty-five women were selected for the first group, 158 for the second, 247 for the third and 160 for the fourth group. Among those, 500 women were selected to perform blood vitamin D measurements after excluding women that were treated with exogenous hormones. Four subjects were not analysed because they had either insufficient sample volume or unreadable data. Five women were excluded because their age at mammography was over 55 years old. Our final analytic sample therefore included 491 premenopausal women. Among these women, 237 had mammography and blood samples obtained during the months of May/June (in Veracruz) and 243 during the months of October/November (in Jalisco). Informed consent was obtained from all participants and the study was approved by the Research, Biosafety and Ethics Committee at the National Institute of Public Health in Mexico, and by the IARC Ethics Committee.

### Mammographic density

A radiology technician performed mammography using the Giotto Image M (Internazionale Medico Scientifica, Bologna, Italy) in Jalisco and the Hologic Lorad M-III (Hologic, Bedford, MA) in Veracruz. Mammograms were developed using the Agfa CP1000 (Agfa-Gevaert Group, Belgium) developer. Craniocaudal views were taken on each breast. An Astra 2400S scanner (Umax, Fremont, CA) was used to digitize the mammograms. A single observer measured MD on the left craniocaudal view using Mamgr, a computer-assisted program developed at the Department of Epidemiology and Population Health, London School of Hygiene and Tropical Medicine [35, 36]. This thresholding software measures the total area as well as the total dense area on a mammogram. Percent MD is automatically calculated as the percent of “dense” pixels within the total breast area. Non-dense area was calculated by subtracting the dense area from the total breast area. Absolute dense and non-dense area values are converted to cm^2^ according to the pixel size used in the digitisation. In a reliability study of 100 ESMaestras mammograms, the intraclass correlation coefficient between MD measurements evaluated using the Mamgr software versus the Cumulus program developed at the University of Toronto was 0.87. In 108 duplicate mammograms, the intra-observer intraclass correlation was 0.84.

### Laboratory assays

#### 25(OH)D Assessment

Serum 25(OH)D2 and 25(OH)D3 levels were measured at Heartland Assays LLC (Ames, Iowa) using a liquid chromatography/tandem mass spectrometry (LC/MS/MS) method as previously described [37]. The average intra-assay coefficient of variation was 5.8% for 25(OH)D3, and 12.6% for 25(OH)D2. Only the analyses for serum 25(OH)D3, the primary exposure variable of interest, are presented because only 24 participants (4.8%) had detectable 25(OH)D2 concentrations above the limit of assay sensitivity of 1.56 ng/ml.

#### Hormone analyses

Analyses were performed on never-thawed serum samples continuously stored at −80°C as previously reported [38]. In brief, serum IGF-I and IGFBP3 concentrations were measured by immunoradiometric assays by Beckmann Coulter (Marseille, France) at the laboratory of hormone analyses, Biomarkers Group, International Agency for Research on Cancer (Lyon, France). The intra-assay and inter-assay coefficients of variations were 0.8% and 4.2%, respectively, for a concentration of 19.5 nmol/l for IGF-I, and 1.3% and 3.0%, respectively, for a concentration of 125 nmol/l for IGFBP-3.

### Statistical analysis

Means and standard deviations (SDs), or percentages of selected baseline characteristics of the study population were estimated across predefined categories of serum 25(OH)D3 [39]. Chi-square and ANOVA tests were used to determine whether the distribution of the selected BC risk factors differed across categories of serum 25(OH)D3. Multivariable adjusted Spearman partial correlation coefficients were performed to investigate the association between serum 25(OH)D3 (ng/ml) and other continuous variables including age (years), body mass index (BMI, kg/m2), waist to hip ratio (WHR), and total physical activity (metabolic equivalent of energy expenditure, MetS per week). Multivariable linear regressions were used to estimate the association of different MD measures (percent MD, dense or non-dense areas) with serum 25(OH)D3. Based on population distribution, the quartiles (25th percentile, 50th percentile, 75th percentile) of percent MD that divide the MD set into four equal group were estimated (MD ≤ 22.6%, 22.6–37.7%,; 37.5–51.9%, and > 51.9%). A woman was considered having low MD if she was in the lower quartile (MD ≤ 22.6%), and high MD if she was in the upper quartile (MD >51.9%). A subset of women was selected based on whether they had low or high percent MD, and 25(OH)D3 concentrations were compared by multivariable logistic regressions. Restricting our analysis to these women allowed for a better discrimination between women at low and high BC risk. Additionally, different approaches to nonlinear modelling were used to explore the association between serum 25(OH)D3 and MD: 1) local polynomial regression (LOESS), which is a smoothing method that essentially summarizes the association between outcome and exposure by fitting a multitude of regression models to adjacent subsets of the data [40]; 2) fractional polynomial modelling, which fit models using various transformations of the predictor for which a non-linear association with the outcome is assumed [41]. Final multivariable models were adjusted for age (continuous), age at menarche (continuous), BMI (continuous), physical activity (continuous), and region/season. Serum 25(OH)D3 concentrations were used as continuous variable, or categorical variables (2 categories (≤ and > median*)*, and predefined categories [39]). Separate analyses were performed adjusting for and then stratifying by BMI (< and ≥ median). The multiplicative interaction between serum 25(OH)D3 and BMI was tested by including a cross-product term in the multivariable model. Stratified analyses and tests for interactions were used to further evaluate possible effect modification of IGF1 and IGFBP3 on the association between serum 25(OH)D3 and percent MD. The Sobel-Goodman mediation method [42, 43] was used to assess the mediating role played by IGF1 and IGFP3 in the associations between 25(OH)D3 and percent MD, and to estimate the proportion of the effect that is mediated (%). Bias-corrected CIs for the percentage mediation were obtained through bootstrap techniques with 1,000 replications [44]. All statistical tests were two-sided and *P*-values < 0.05 were considered significant. All statistical analyses were conducted using STATA (version 11).

## Results

Baseline characteristics of the study population across predefined categories of serum 25(OH)D3 are presented in Table 1. The mean age at recruitment of the 491 premenopausal women was 42.9 ± 3.7 years. Overall, the mean serum 25(OH)D3 levels was 28.12 ± 6.93 ng/ml. Forty one (8%) women had 25(OH)D3 levels in the deficient range (< 20 ng/ml), 265 (54%) were in the insufficient range (20–29 ng/ml) and 185 (38%) were in the sufficient range (≥ 30 ng/ml). Statistically significant differences in physical activity (P < 0.001), non-dense area (P = 0.005), and breastfeeding (P = 0.011) were observed across the three categories of 25(OH)D3. No significant differences in age at menarche, age at mammography, age at 1^st^ full term pregnancy, parity, percent MD, dense area, region, BMI, alcohol intake, use of hormonal contraceptive, FHBC and socio economic status were observed (Table 1).

**Table 1:** Characteristics of the study population across predefined cut-points of serum 25(OH)D3.

	Serum 25(OH)D3 (ng/ml)			
Characteristic	< 20 (n = 41)	20–29 (n = 265)	≥ 30 (n = 185)	P-value
**Means ± SD**				
Age at mammography (years)	43.6±4.2	43.1±3.6	42.6±3.6	0.457
Age at menarche (years)	12.2±1.2	12.5±1.4	12.5±1.5	0.343
Age at 1^st^ full term pregnancy (years)	24.8±4.4	24.8±4.3	24.6±4.8	0.407
parity	1.77±1.4	2.13±1.2	2.16±1.2	0.261
Total physical activity (MetS per week)	24.3±16.7	25.9±19.5	28.7±25.0	<0.001
Percent MD	36.4±18.6	37.6±17.5	37.1±17.1	0.778
Dense area (cm^2^)	46.8±30.6	50.5±34.6	48.1±31.8	0.343
Non dense area (cm^2^)	83.4±45.4	82.1±39.9	78.7±33.1	0.005
**Frequency n (%)**				
Region				
Jalisco	26(10.6)	126(51.6)	92(37.7)	0.167
Veracruz	15(6.1)	139(56.3)	93(37.6)	
Body mass index (kg/m^2^)				
< 30 kg/m^2^	22(7.1)	161(51.7)	128(41.2)	0.076
≥ 30 kg/m^2^	19(10.5)	104(57.8)	57(31.7)	
Breastfeeding				
Never	10(19.2)	25(48.1)	17(32.7)	0.011
Ever	31(7.1)	240(54.7)	168 (38.3)	
Alcohol intake				
No	14(9.9)	65(46.1)	62(44.0)	0.085
Yes	27(7.7)	200(57.1)	123(35.1)	
Ever use of hormonal contraceptive				
No	24(10.5)	124(54.1)	81(35.4)	0.364
Yes	17(6.9)	133(54.1)	96(39.0)	
Missing	0(0)	8(50)	8(5)	
Family history of breast cancer				
No	38(8.1)	252(53.7)	179(38.2)	0.462
Yes	3(13.6)	13(59.1)	6(27.3)	
Socio economic status				
Low	5(7.1)	39(55.7)	26(37.1)	0.519
Medium	19(10.3)	102(55.1)	64(34.6)	
High	15(8.9)	89(52.9)	64(38.1)	
Missing	2(2.9)	35(51.5)	31(45.6)	

P value based on chi square and ANOVA tests.

Table 2 shows Spearman partial correlation coefficients and P-values for the correlations between serum 25(OH)D3 levels and other factors. Serum 25(OH)D3 levels were inversely correlated with BMI (r = −0.109, P = 0.019) and directly related with total physical activity (r = 0.095, P = 0.035). No statistically significant correlations were found between serum 25(OH)D3 levels and others factors (age, WHR, dietary calcium, IGF1 and IGFBP3) (Table 2).

**Table 2:** Correlation between serum 25(OH)D3 and other risk factors.

Characteristic	n	Spearman correlation coefficient ^a^	P*-*value ^b^
Age (years)	491	-0.045	0.311
BMI (kg/m^2^)	454	-0.109	0.019
WHR	430	0.017	0.723
Total physical activity (MetS/week)	490	0.095	0.035
Dietary calcium intake (mg/day)	474	-0.041	0.361
IGF1 (ng/ml)	474	0.016	0.719
IGFBP3 (ng/ml)	474	0.082	0.069

BMI body mass index, WHR waist hip to hip ratio, MetS, metabolic equivalents of energy expenditure.

^a^ Spearman correlation coefficient adjusted for age, total physical activity and BMI.

^b^ P*-*value.

In the adjusted multivariate linear regression, no significant associations were observed between serum 25(OH)D3 levels (in continuous) and different measures (percent MD, dense area and non-area) overall (Table 3). In stratified analyses according to the median of serum 25(OH)D3 levels (<27.3 ng/ml and ≥27.3 ng/ml), higher serum 25(OH)D3 levels (≥ 27.3 ng/ml) were marginally related to percent MD (β = −0.38, P = 0.059) (Table 3). When stratified by median BMI (< and ≥ 27.4 kg/m^2^), a borderline statistically significant inverse association was found between higher serum 25(OH)D3 levels (> median) and MD only among women with BMI below the median (β = −0.52, P = 0.047) compared to those with BMI above or equal to the median (β = −0.14, P = 0.699) (not shown). In the analyses stratified by the WHO cut-off point for normal BMI (< 25 kg/m^2^) and overweight BMI (≥ 25 kg/m^2^), no significant heterogeneity association was observed, although in overweight women (BMI ≥ 25 kg/m^2^) the association with non-dense area was significant (S1 Table).

**Table 3:** Multivariable linear regression estimates of percent MD (%), dense area (cm^2^), and non-dense area (cm^2^).

Characteristic	Percent Mammographic density (%)		Dense area (cm^2^)		Non-dense area (cm^2^)	
	β coefficient (95% CI)	P-value	β coefficient(95% CI)	P-value	β coefficient (95% CI)	P-value
Serum 25(OH)D3 (ng/ml)						
Overall	-0.02(-0.24, 0.20)	0.840	0.07(-0.37, 0.50)	0.754	-0.13(-0.53, 0.28)	0.537
< median (27.3 ng/ml)	0.25(-0.42, 0.91)	0.469	1.02(-0.27, 2.31)	0.121	-0.2(-1.45, 1.04)	0.747
≥ median(27.3 ng/ml)	-0.39(-0.81, 0.01)	0.059	-0.42(-1.21, 0.37)	0.295	0.34(-0.36, 1.04)	0.338
Predefined categories						
< 20	1 (ref)		1 (ref)		1 (ref)	
20–30	1.98(-3.67, 7.63)	0.491	7.03(-3.92, 17.99)	0.208	0.05(-10.23, 10.33)	0.992
≥ 30	1.26(-4.67, 7.19)	0.676	6.38(-5.11, 17.88)	0.276	-0.71(-11.50, 10.08)	0.897
Region						
Jalisco	-0.05(-0.35, 0.24)	0.725	0.05(-0.52, 0.63)	0.850	0.19(-0.46, 0.50)	0.937
Veracruz	0.05(-0.27, 0.37)	0.759	0.19(-0.44, 0.82)	0.550	-0.07(-0.73, 0.58)	0.827
Age	-1.73(-3.63, 0.17)	0.074	-2.97(-6.67, 0.73)	0.116	0.46(-3.00, 3.93)	0.793
Age at menarche	0.41(-0.59, 1.41)	0.423	0.63(-1.31, 2.59)	0.523	-0.26(-2.09, 1.57)	0.779
Body mass index (kg/m^2^)	-0.52(-0.80, -0.23)	<0.001	1.42(0.87, 1.97)	<0.001	4.32(3.81, -4.83)	<0.001
Total physical activity (MetS per week)	0.008(-0.06, 0.07)	0.812	-0.03(-0.16, 0.09)	0.627	-0.04(-0.16, 0.08)	0.533

Multivariable models were adjusted for age, age at menarche, body mass index, total physical activity and region/season.

No statistically significant associations were observed when we assessed serum 25(OH)D3 levels as predefined categories (<20, 20–29, and ≥30 ng/ml) (Table 3). There were no significant differences in associations between serum 25(OH)D3 and region (P interaction = 0.779).

**Fig 1:**
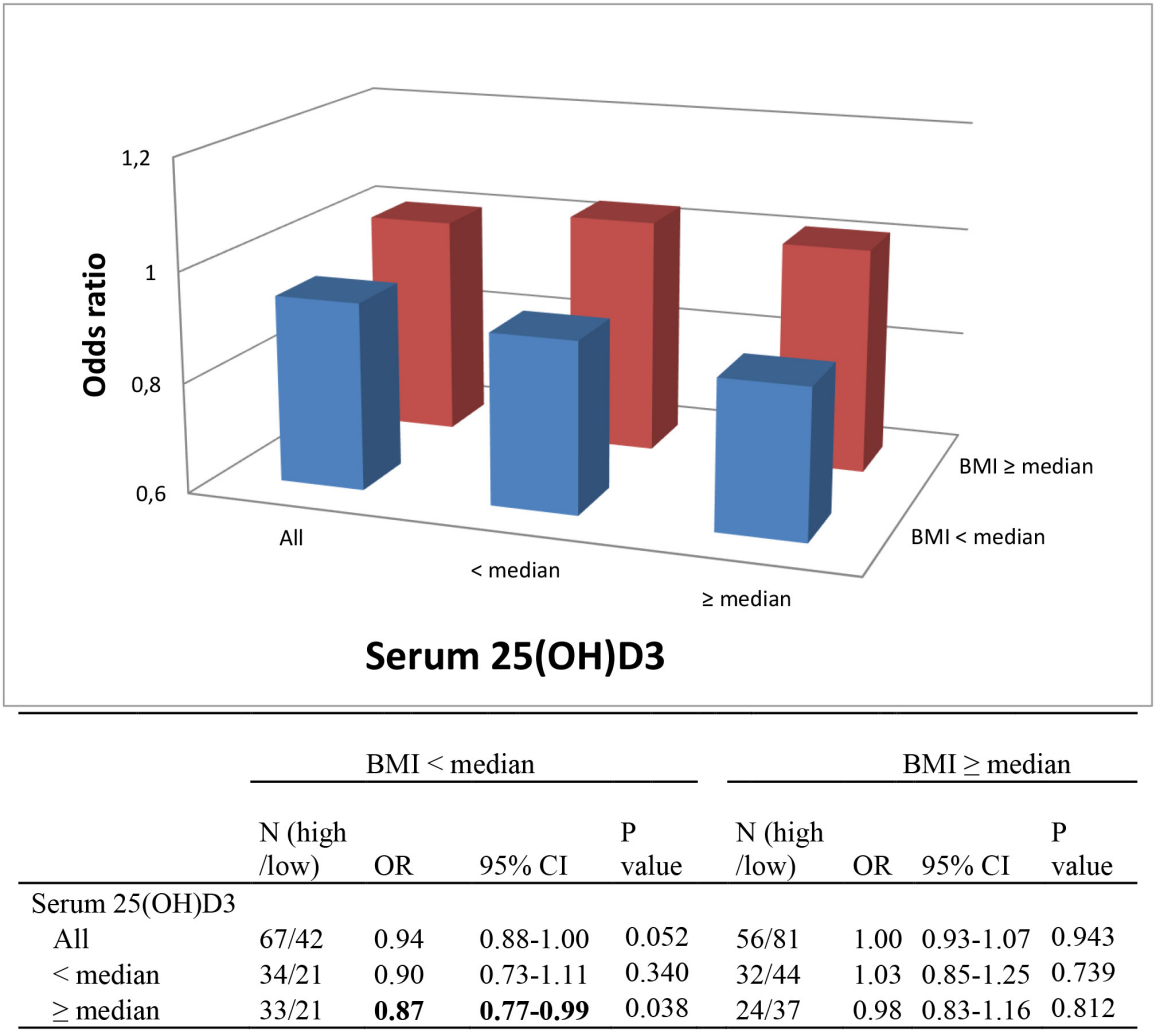
Association of serum 25(OH)D3 and percent MD, stratified by BMI. Based on population distribution, the quartiles (25th percentile, 50th percentile, 75th percentile) of percent MD that divide the MD set into four equal group were estimated (MD ≤ 22.6%, 22.6–37.7%,; 37.5–51.9%, and > 51.9%). A subset of women was selected based on whether they had low percent MD (≤ 22.6%) or high percent MD (> 51.9%), and 25(OH)D3 concentrations were compared by multivariable logistic regressions. Multivariable models were adjusted for age, age at menarche, total physical activity and season of blood draw. Median BMI = 27.4 kg/m2, median serum 25(OH)D = 27.3ng/ml. N (high/low): number (high MD/ low MD), OR: odds ratio; 95% CI 95% confidence interval.

In the analyses restricted to women classified as having low MD (≤22.6%) and high MD (>51.9%), there was limited evidence of an inverse association between high serum 25(OH)D3 and MD. The odds ratio (OR) of having high MD for women with serum 25(OH)D3 above the median was 0.91(95%CI: 0.83–0.99) (not shown). This inverse association was significant only among women with BMI below the median (OR = 0.87, 95%CI: 0.78–0.98) (Fig 1). However there was no significant interaction between serum 25(OH)D3 (continuous) and BMI (continuous) (P interaction = 0.283). This could be due to the small sample size.

**Fig 2:**
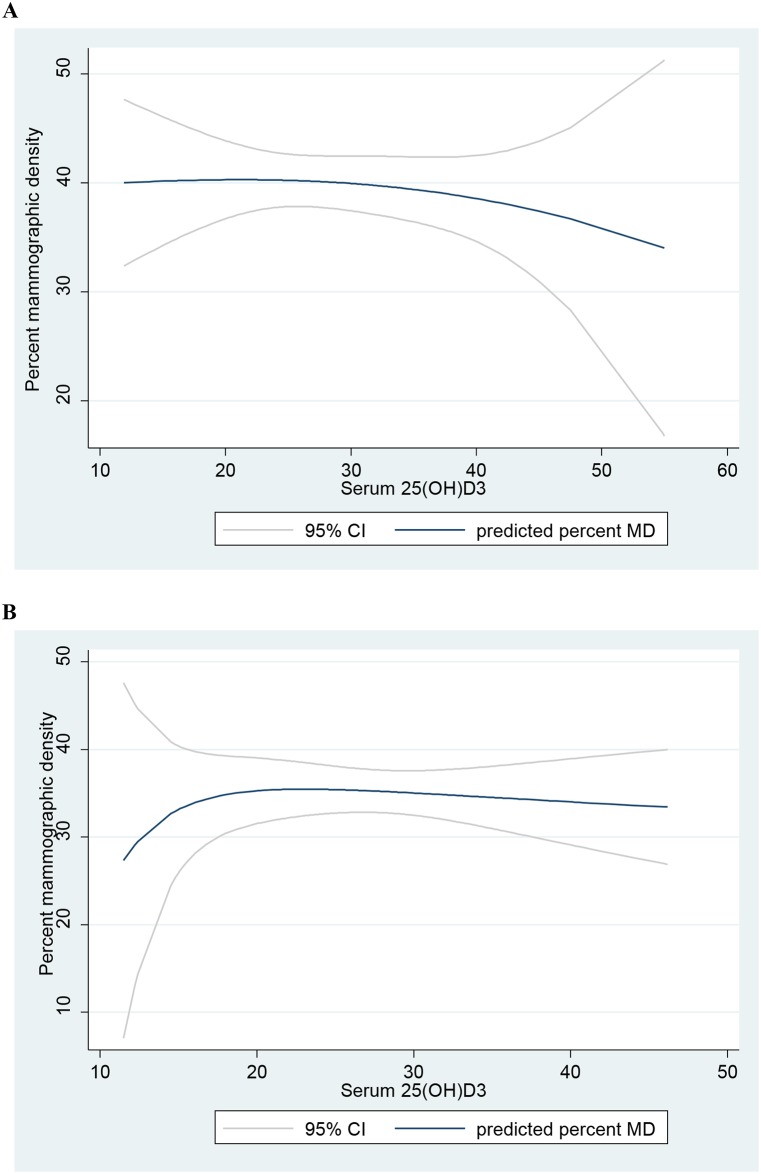
Fractional polynomial modelling of the association of serum 25(OH)D3 (ng/ml) with percent mammography density (MD). A model with 95%CI among women with: A) BMI < median (< 27.4 kgm^2^), B) BMI ≥ median (≥ 27.4 kgm^2^).

In non-linear models including fractional polynomial modelling, there was a trend of an inverse the association between serum 25(OH)D3 levels and MD only among women with BMI < median (Fig 2). However the test of non-linearity was not significant.

Finally, we investigated whether IGF1 and IGFBP3 modified the relation of serum 25(OH)D3 with MD, and no statistically significant interactions were found (P interaction were 0.764 and 0.398, respectively for IGF1 and IGFBP3, S2 Table). In the mediation analyses, no evidence of the indirect effects of serum 25(OH)D3 on MD through IGF1 and IGFBP3 was observed (all P value > 0.05) (S2 Table).

## Discussion

In this study, we observed a non-significant inverse association between serum 25(OH)D3 levels and MD overall. However, in the analyses restricted to women classified as having low MD and high MD, we observed an inverse association between high serum 25(OH)D3 and MD although of borderline significance. When stratified by BMI, this inverse association appeared statistically significant only among women with BMI below the median.

Our finding of non-significant association overall between serum 25(OH)D3 levels and MD among premenopausal women is consistent with results from several studies [21–23, 25]. More recently the study by Crew et al on 195 women aged 40–60 years found no association between serum 25(OH)D and different measures of MD, however [22]. In a recent Korean study, the authors reported a small but statistically significant association between serum 25(OH)D and MD in correlation analyses, but no significant association was observed in the multivariable regression analyses [23]. A cross-sectional study by Chai et al., showed that serum 25(OH)D was not associated with MD among 182 premenopausal women [21]. Indeed, the associations between vitamin D and MD are not fully understood, as previous findings have shown inconsistent results. Some studies reported no significant associations [21, 23, 25] with lower MD among both pre- and post-menopausal women [19, 24]. In contrast to our study, Brisson et al. reported that changes in circulating vitamin D (25(OH)D) were inversely associated with changes in MD with a lag time of about four months [18]. This study showed the importance of the lag time that could be a critical concept when assessing the relation of changes in circulating 25(OH)D to changes in MD. This may explain the lack of significant association observed overall in our study. However, our blood sample collections were done over 4 months in two regions (May/June in Jalisco and October/November in Veracruz) do not allow a lag time analysis.

Comparing high MD (highest quartile) and low MD (lowest quartile) we observed a significant inverse association between serum 25(OH)D3 and MD among the group with lower BMI (< median) and higher serum 25(OH)D3 (> median). This suggests that the effect of vitamin D may be observed after a certain level in the blood only in women with BMI below the median. The putative inverse relationship between vitamin D and obesity was firstly described by Rosenstreich et al. in 1971 [45]. The notion that vitamin D level is related to obesity is also consistent with a recent meta-analysis of twelve studies, which reported a pooled relative risk of 1.52 (95% CI: 1.33–1.73) for risk of vitamin D deficiency (< 50 nmol/L (equivalent to 15.7 ng/ml)) in obese people (BMI > 30 kg/m^2^) [46]. Likewise, although Kuhn et al. reported that overall levels of serum 25(OH)D were not associated with the risk of BC, they found a non-significant inverse association among women with BMI < 25 kg/m^2^ (OR = 0.83; 95% CI: 0.67–1.03, p = 0.09) [47].

Furthermore, several studies reported different strength of the association between vitamin D and MD according to some circulating growth factors levels [26, 48]. This was shown in the study of Diorio et al. which reported a stronger inverse association between MD and dietary vitamin D level in women with higher IGF-1 and lower IGFBP3 levels than in those with low levels, suggesting that the association between vitamin D, calcium and MD may be limited to premenopausal women, who have higher levels of calcium, IGF-1 and IGFBP-3 [26]. In contrast, our results showed no effect modification by these biomarkers.

The main classical roles of vitamin D involve the regulation of calcium metabolism and skeletal remodelling [11, 12]. Vitamin D has also significant anticancer effects including inhibition of proliferation, induction of differentiation, and promotion of apoptosis in breast cells [49, 50]. In addition, vitamin D actions involve: transcriptional repression of aromatase via promoter II in BC cells and surrounding adipose tissue; decrease in prostaglandin E2 (PGE2), a major stimulator of aromatase transcription in BC cells; transcriptional repression of ER in BC cells to block oestrogen stimulus [14]. The protective effects of vitamin D have been shown to function mainly through the vitamin D receptor (VDR) present in breast cells [16, 48]. Vitamin D can also influence MD by indirect effects due to overlap with other pathways [16, 51]. Some studies have suggested that the effect of Vitamin D in the breast might result from its effect on the insulin growth factor signalling pathway [11, 19]. Vitamin D has been shown to stimulate and enhance the expression of IGFBP-3 [16, 29]. In contrast, it inhibits the mitogenic effect of IGF1, attenuates the antiapoptotic effect of IGF1, and down regulates the expression of IGF1 receptors [29, 52, 53]. However in our mediation analyses, we did not observe any evidence of the indirect effects of serum 25(OH)D3 on MD through IGF1 and IGFBP3.

The current study had several strengths, including the use of serum 25(OH)D3 concentrations, which is the best biomarker reflecting the total body vitamin D levels and long-term vitamin D. The blood draws and mammograms were taken on the same date, and all mammograms were evaluated with a computer assisted method. We adjusted for potentials confounding factors and account for region/season. This is the first study conducted among Mexican women, however some limitations should be considered. We used a single measure of vitamin serum levels, which may conduct to a possible vitamin D exposure misclassification since circulating vitamin D is prone to seasonal variability. However, a previous study suggested that serum 25(OH)D concentration at a single time point may be a useful biomarker of long-term vitamin D status [54]. Another limitation is the potential misclassification of participants’ vitamin D status, due to excluding serum 25(OH)D2 concentrations. However this should be minimal, since unlike vitamin D3, vitamin D2 is present mostly in fungus-/yeast-derived products; thus, its contribution to overall vitamin D status is negligible. In addition, vitamin D3 is the most utilized form of vitamin D in clinical trials [55].

In conclusion, although, no significant association was observed between serum 25(OH)D3 and percent MD in the overall population, our study supports an inverse association between higher serum 25(OH)D3 levels (> median) and MD in premenopausal women with BMI below the median women. It is possible that the impact of 25 (OH)D3 is observed only after a certain threshold in the blood. Indeed, the Endocrine Society has stated that the desirable serum concentration of 25(OH)D was >30 ng/ml to maximize its effect on calcium, bone, and muscle metabolism [39]. More research is needed to understand this association and to see whether vitamin D supplementation may play a preventive role in BC.

## Supporting Information

S1 Table Multivariable linear regression estimates of percent MD (%), dense area (cm^2^), and non-dense area (cm^2^), stratified by normal (< 25 kg/m^2^) and overweight BMI (≥ 25 kg/m^2^). Multivariable models were adjusted for age, age at menarche, body mass index, total physical activity and region/season. Serum 25(OH)D3 concentrations were used as continuous variable, or categorical variables (2 categories: ≤ and > median*)*, and predefined categories.
(DOCX)

S2 Table Multivariate Sobel–Goodman mediation tests and bias-corrected confidence interval. CI, confidence interval. Multivariable models were adjusted for age, age at menarche, body mass index, total physical activity and region/season. Tests for interactions were used to further evaluate possible effect modification of IGF1 and IGFBP3 on the association between serum 25(OH)D3 and percent MD. The Sobel-Goodman mediation method was used to assess the mediating role played by IGF1 and IGFP3 in the associations between 25(OH)D3 and percent MD, and to estimate the proportion of the effect that is mediated (%). Bias-corrected CIs for the percentage mediation were obtained through bootstrap techniques with 1,000 replications.
(DOCX)
