# Supplementary material for: Serum 25–Hydroxyvitamin D3 and Mammography Density among Mexican Women
Source: PLoS One. 2016 Aug 26;11(8):e0161686. doi: 10.1371/journal.pone.0161686 (PMC5001725; doi:10.1371/journal.pone.0161686)
Supplement: S1 Table — Multivariable models were adjusted for age, age at menarche, body mass index, total physical activity and region/season. Serum 25(OH)D3 concentrations were used as continuous variable, or categorical variables (2 categories: ≤ and > median), and predefined categories. (DOCX) [file pone.0161686.s001.docx]

**S Table 1. Multivariable linear regression estimates of percent MD (%), dense area (cm²), and non-dense area (cm²), stratified by normal (< 25 kg/m^2^) and overweight BMI (≥ 25 kg/m^2^)**

|  | BMI < 25 kg/m^2^ | | BMI ≥ 25 kg/m^2^ | |
| --- | --- | --- | --- | --- |
| Characteristic | β coefficient (95% CI) | P-value | β coefficient (95% CI) | P-value |
| **Percentage density (%)** |  |  |  |  |
| Serum 25(OH)D3 (ng/ml) | |  |  |  |
| Continuous | -0.23(-0.64, 0.18) | 0.275 | 0,08(-0.18, 0.35) | 0.525 |
| < median | -0.006(-1.33, 1.31) | 0.992 | 0.52(-0.23, 1.28) | 0.177 |
| ≥ median | -0.65(-1.39, 0.09) | 0.087 | -0.20(-0.73, 0.32) | 0.442 |
| Predefined categories | |  |  |  |
| < 20 | 1.00 |  | 1.00 |  |
| 20-30 | 2.75(-8.82, 14.3) | 0.639 | 2.45(-4.17, 9.08) | 0.467 |
| ≥ 30 | 0.99(-10.9, 12.9) | 0.869 | 2.33(-4.65, 9.32) | 0.511 |
| **Dense area (cm²)** | |  |  |  |
| Serum 25(OH)D3 (ng/ml) | |  |  |  |
| Continuous | -0.14(-0.72, 0.44) | 0.646 | -0.04(-0.60, 0.50) | 0.868 |
| < median | 0.70(-1.15, 2.57) | 0.450 | 1.00(-0.61, 2.61) | 0.224 |
| ≥ median | -0.78(-1.81, 0.24) | 0.134 | -0.09(-1.16, 0.97) | 0.862 |
| Predefined categories | |  |  |  |
| < 20 | 1.00 |  | 1.00 |  |
| 20-30 | 6.45(-9.72, 22.6) | 0.431 | 4.83(-9.01, 18.6) | 0.493 |
| ≥ 30 | 4.61(-12.1, 21.3) | 0.587 | 2.40(-12.1, 16.9) | 0.746 |
| **Non-dense area (cm²)** | |  |  |  |
| Serum 25(OH)D3 (ng/ml) | |  |  |  |
| Continuous | 0.22(-0.35, 0.80) | 0.445 | -0.73(-1.35, -0.10) | 0.022 |
| < median | 0.37(-1.50, 2.24) | 0.693 | -1.31(-3.21, 0.59) | 0.177 |
| ≥ median | 0.38(-0.63, 1.40) | 0.449 | 0.13(-1.02, 1.29) | 0.815 |
| Predefined categories | |  |  |  |
| < 20 | 1.00 |  | 1.00 |  |
| 20-30 | -4.85(-20.8, 11.1) | 0.549 | -5.21(-20.8, 10.4) | 0.512 |
| ≥ 30 | -3.64(-20.2, 12.9) | 0.664 | -11.2(-27.7, 5.25) | 0.181 |

Multivariable models were adjusted for age, age at menarche, body mass index, total physical activity and region/season. Serum 25(OH)D3 concentrations were used as continuous variable, or categorical variables (2 categories : ≤ and > median), and predefined categories
