# Supplementary material for: Serum 25–Hydroxyvitamin D3 and Mammography Density among Mexican Women
Source: PLoS One. 2016 Aug 26;11(8):e0161686. doi: 10.1371/journal.pone.0161686 (PMC5001725; doi:10.1371/journal.pone.0161686)
Supplement: S2 Table — CI, confidence interval. Multivariable models were adjusted for age, age at menarche, body mass index, total physical activity and region/season. Tests for interactions were used to further evaluate possible effect modification of IGF1 and IGFBP3 on the association between serum 25(OH)D3 and percent MD. The Sobel-Goodman mediation method was used to assess the mediating role played by IGF1 and IGFP3 in the associations between 25(OH)D3 and percent MD, and to estimate the proportion of the effect that is mediated (%). Bias-corrected CIs for the percentage mediation were obtained through bootstrap techniques with 1,000 replications. (DOCX) [file pone.0161686.s002.docx]

**S Table 2. Multivariate Sobel–Goodman mediation tests and bias-corrected confidence interval**

|  | Serum 25(OH)D3 | | |
| --- | --- | --- | --- |
| Mediator | β coefficient and bias corrected CI | P-value | P-interaction |
| IGF1 | 0.0007(-0.008, 0.019) | 0.780 | 0.764 |
| IGFBP3 | 0.0023(-0.011, 0.031) | 0.760 | 0.398 |

CI, confidence interval. Multivariable models were adjusted for age, age at menarche, body mass index, total physical activity and region/season. Tests for interactions were used to further evaluate possible effect modification of IGF1 and IGFBP3 on the association between serum 25(OH)D3 and percent MD. The Sobel-Goodman mediation method was used to assess the mediating role played by IGF1 and IGFP3 in the associations between 25(OH)D3 and percent MD, and to estimate the proportion of the effect that is mediated (%). Bias-corrected CIs for the percentage mediation were obtained through bootstrap techniques with 1,000 replications.
